# Thermal and Dielectric Investigations of Polystyrene Nanoparticles as a Viable Platform—Toward the Next Generation of Fillers for Nanocomposites

**DOI:** 10.3390/polym15132899

**Published:** 2023-06-30

**Authors:** Mihai Asandulesa, Ana-Maria Solonaru, Ana-Maria Resmerita, Andrei Honciuc

**Affiliations:** “Petru Poni” Institute of Macromolecular Chemistry, 41A Grigore Ghica Voda Alley, 700487 Iasi, Romania; solonaru.anamaria@icmpp.ro (A.-M.S.);

**Keywords:** polystyrene, nanoparticles, broadband dielectric spectroscopy, differential scanning calorimetry, glass transition temperature

## Abstract

Nanoparticles are often used as fillers for enhancing various properties of polymer composites such as mechanical, electrical, or dielectric. Among them, polymer nanoparticles are considered ideal contenders because of their compatibility with a polymer matrix. For this reason, it is important that they are synthesized in a surfactant-free form, to obtain predictable surface and structural properties. Here, we synthesized a series of polystyrene nanoparticles (PS NPs), by emulsion polymerization of styrene, using varying amounts of divinylbenzene as a crosslinking agent and sodium 4-vinylbenzenesulfonate as a copolymerizing monomer surfactant—“surfmer”. Using “surfmers” we obtained surfactant-free nanoparticles that are monodisperse, with a high degree of thermal stability, as observed by scanning electron microscopy and thermogravimetric investigations. The prepared series of NPs were investigated by means of broadband dielectric spectroscopy and we demonstrate that by fine-tuning their chemical composition, fine changes in their dielectric and thermal properties are obtained. Further, we demonstrate that the physical transformations in the nanoparticles, such as the glass transition, can be predicted by performing the first derivative of dielectric permittivity for all investigated samples. The glass transition temperature of PS NPs appears to be inversely correlated with the dielectric permittivity and the average diameter of NPs.

## 1. Introduction

Nanotechnology is at the forefront of current research and development, being deeply embedded in a wide range of disciplines, from biotechnology to electronics, robotics, and artificial intelligence [1,2,3]. With these advances, the field of polymer nanocomposites has sparked great interest in the scientific community and is considered to be the foundation for generating materials with enhanced mechanical, electric, or dielectric properties, etc. The most common type of nanocomposites consists of dispersing nanoparticles into a polymer matrix; thus, nanotechnology enables a rational design of nanocomposite materials, where operating small changes in nanoparticle structures can change their interaction with the polymer matrix, which in turn produces a predictable variation in form, function, and properties of the nanocomposite [4,5,6]. There are numerous methods to prepare polymer nanocomposites, such as in situ polymerization, melt extrusion, and solution dispersion [7,8]. The most common nanoparticles employed in the preparation of polymer nanocomposites are in the form of quantum dots, nanofibrils, nanosheets, and nanotubes. To prepare a quality composite, the first necessary conditions are to ensure an excellent dispersion of nanoparticles in the polymer, as well as an excellent compatibility between the components [9,10]. This critical condition related to ideal mixing and efficient reinforcement of polymer nanocomposites is still problematic and thus remains a challenge and a focus for ongoing research [11,12].

Polystyrene nanoparticles have various distinguishing characteristics that make them suitable for a variety of applications and among other things may be useful in medical applications such as imaging and drug delivery [13,14], materials science, electronics [15], and the preparation of new classes of polymer nanocomposites. To produce a high-quality polymer nanocomposite, the PS NPs must fulfill the first condition, namely, to be highly dispersible in the polymer matrix. To achieve this, PS NPs must be prepared with good control over their surface chemical properties, such as surface polarity, which ensures compatibility with the polymer matrix; must have good thermal stability; and must be prepared in pure form. We have previously shown [16] that to prepare high-quality PS NPs via the emulsion polymerization technique, surfactants used in classical synthesis must be eliminated; instead, we proposed using surfactant monomers “surfmers”, which disappear after the polymerization reaction, such as sodium 4-vinylbenzenesulfonate NaVBS. The proportion of the “surfmers” used in the NPs composition affects their surface properties but also their sizes. A second component greatly affecting their properties is divinylbenzene (DVB), influencing their crosslinking degree, which affects the NPs’ size and thermal resistance. We have previously demonstrated that NaVBS and DVB are indeed the key components in fine-tuning surface and intrinsic mass properties of the PS NPs when preparing the next generation of amphiphiles, the Janus nanoparticles (JNPs), by growing a polar component onto the spherical PS NPs [17,18]. As previously mentioned, a great disadvantage in the preparation of polymer nanocomposites is the limited dispersibility of the semiconducting fillers and, thus, their poor dispersion in polymer matrices. In this regard, because snowman-shaped amphiphilic JNPs prepared from PS NPs using seeded emulsion polymerization exhibited an inherent polar/nonpolar duality [16] that can be tuned to make them more compatible with any polymer matrix they emerge as ideal candidates in the preparation of future nanocomposites; further, Mihali et al. demonstrated that by adjusting the amount of NaVBS and DVB in the composition of PS NPs can produce a whole series of JNPs with different dimensions, degrees of phase separation, and polarity balance [19,20]. While the effects of adjusting the amounts of NaVBS and DVB are well understood in terms of how the physicochemical properties, function, and structure are affected in the final PS NPs and how the adjustment of the amounts of these two components affects the further construction of JNPs, in this work we propose to understand how their dielectric and thermal properties change. Currently, to the best of our knowledge, this is the first approach to understanding the impact of various components used in the synthesis of seed PS NPs and especially that of the crosslinking agent DVB and that of “surfmer” NaVBS.

Broadband dielectric spectroscopy (BDS) is a non-invasive experimental technique that characterizes the fluctuations of electric dipole moments within the molecules and the diffusion of charge carriers through a material backbone [21,22]. These measurements usually cover a vast frequency range and may be performed in a large range of temperatures, allowing investigation of molecular relaxation processes of different materials [23,24]. In this regard, BDS offers high accuracy and reproducibility for the evaluation of the glass transition temperature of polymers, polymer blends, co-networks, and composites [25,26,27,28]; moreover, it was previously reported that BDS is a very sensitive procedure that may assess the density fluctuations, and, consequently, emphasize the phase transitions of investigated samples [29]. In some polymeric systems, principally in the case of heterogeneous materials, dielectric spectroscopy recognizes the most common effects of the phenomenon of electrical polarization: (i) electrode polarization (i.e., agglomeration of charge carriers at the interface between the sample surfaces and the electrodes employed for dielectric measurements), and (ii) Maxwell–Wagner–Sillars interfacial polarization (i.e., changes in the local conductivity at boundaries between the two phases with different conductivities) [30].

In this paper, a series of four spherical PS NPs were prepared by emulsion polymerization of styrene in the presence of small amounts of NaVBS “surfmer”. The presented research includes the effects of the DVB crosslinking agent and NaVBS on the size and uniformity and their dielectric properties as investigated with the BDS technique. The glass transition temperature of nanoparticles was assessed from the first derivative of the dielectric permittivity parameter. Finally, a direct connection between the crosslinker and “surfmer” with the glass transition and permittivity of prepared nanoparticles was found.

## 2. Materials and Methods

### 2.1. Materials

Styrene (>99%) contains 4-tert-butylcatechol as stabilizer, divinylbenzene (DVB, technical grade 80%) contains monomethyl ether hydroquinone as inhibitor, sodium 4-vinylbenzenesulfonate (NaVBS, >90%), ammonium peroxydisulfate (APS, >98%), aluminum oxide (Al_2_O_3_, ≥98%) were purchased from Sigma-Aldrich (Merck KGaA, Darmstadt, Germany). Styrene and DVB were passed through aluminum oxide to remove the stabilizer before use. Ethanol and methanol were purchased from Chemical Company (Iasi, Romania) and were used as received. Distilled water was used in all experiments and was obtained from a Lauda Puridest PD 2 R GFL Technology (Lauda-Königshofen, Germany) water-distilled system.

### 2.2. Synthesis of Polystyrene Nanoparticles

The typical procedure for the synthesis of PS NPs with different dimensions is as follows: NaVBS and APS (135 mg) were dissolved in 200 mL mixture of distilled water and methanol (*v*/*v* = 9/1) at room temperature. Then, a mixture consisting of styrene and DVB was added to solution, and oxygen in the suspension was further removed under nitrogen atmosphere for 30 min. The polymerization was carried out under stirring at 700 rpm and at 70 °C for 24 h. Finally, the polymerization solution was filtered, and the PS NPs were purified by centrifugation and washed with distilled water and ethanol three times. A series of four PS NPs were synthesized by varying the amount of NaVBS, styrene, and DVB as shown in Table 1.

### 2.3. Characterization Methods

Fourier Transform Infrared (FT-IR) spectra were collected in the Attenuated Total Reflection (ATR) mode with a DIGILAB-FTS 2000 Spectrometer (Bruker, Karlsruhe, Germany). The samples were obtained in the form of dried powder and then deposited on the ATR crystal of the device. The infrared spectra were recorded in the spectral region of 400–4000 cm^−1^.

The morphology of PS NPs was analyzed with a Verios G4 UC Scanning Electron Microscope (SEM) from Thermo Fischer Scientific (Eindhoven, The Netherlands). Prior to SEM investigations, the samples were prepared by drying an aqueous particle suspension on the surface of the aluminum sample holder. Measurements were carried out in high vacuum regime, employing a standard secondary electron detector (Everhart-Thornley detector, ETD) and a beam energy between 4.5 eV and 5.4 eV.

Thermogravimetric (TG) and derivative thermogravimetric (DTG) measurements were carried out with Mettler Toledo TGA-SDTA851e equipment (Mettler Toledo, Greifensee, Switzerland) under a constant nitrogen atmosphere (flow rate of 20 cm^3^/min) and at temperatures between 25 °C and 800 °C (heating rate of 10 °C/min). The Mettler Toledo STARe software (Version 9.10, Giessen, Germany) was employed for TG and DTG curves analysis.

Differential scanning calorimetry (DSC) measurements were performed in nitrogen atmosphere with the Mettler Toledo DSC-1 calorimeter (Mettler Toledo, Greifensee, Switzerland). PS nanoparticles were deposited in alumina crucibles and then two heating–cooling cycles with a fixed rate of 10 °C/min were applied at temperatures between 0 °C and 180 °C. The first cycle was intended to erase the thermal history, while the second heating and subsequent cooling scans were selected for investigation.

The dielectric properties of PS NPs were measured with the broadband dielectric spectrometer (Novocontrol Technologies, Montabaur, Germany). For dielectric measurements, the aqueous NPs suspension was dried and the resulting powder was further compacted (10 ton) with a uniaxial hydraulic press to form circular pellets 0.04 cm thick and 1.3 cm diameter. Finally, the pellets were placed between two gold-coated plate electrodes provided by Novocontrol and introduced into the ZGS active sample cell of the BDS device. The measurements were carried out under a flow of a dry nitrogen atmosphere, excluding the moisture from environment. The alternating electric field was provided by the Alpha-A Analyzer and the temperature was assisted by a Quatro Cryosystem device. The complex dielectric permittivity, ε* = ε′ − iε″, was collected under isothermal conditions, in a frequency window between 0.1 Hz and 1 MHz. The frequency scans were recorded at every 5 °C, at temperatures between −150 °C and 280 °C.

## 3. Results and Discussions

### 3.1. Preparation of Polystyrene Nanoparticles with Different Diameters

**Figure 1:**
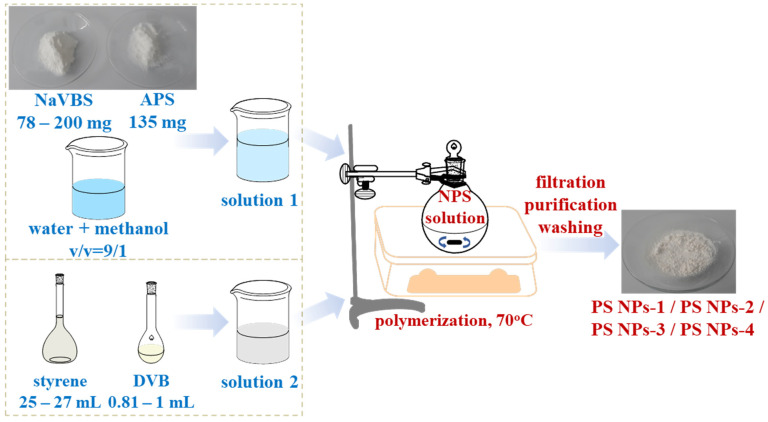
Schematic diagram for the preparation of PS NPs. Representative images of NaVBS, APS, and NPS powders are included.

The current seed PS-NPs are the same as those used in the preparation JNPs in our previous works; thus, investigating their dielectric properties as a function of the composition represents the first step toward the preparation of the next generation of JNPs fillers with adjustable dielectric properties and enhanced dispersibility in aqueous solution as a green platform for the preparation of nanocomposites. The PS NPs were consequently obtained using varying amounts of reactants as follows: (i) styrene was measured between 25 mL and 27 mL; (ii) NaVBS was varied between 78 mg and 200 mg; and (iii) DVB was varied between 0.81 mL and 1 mL, as also given in Table 1. The preparation of nanoparticles is schematically presented in Figure 1. The ratio between reactants is reflected in the diameter of the prepared nanoparticles as well as other physical properties (glass transition, dielectric permittivity). The effect of the synthesis conditions on physical characteristics is discussed in the final section of the manuscript.

### 3.2. Chemical Structure

**Figure 2:**
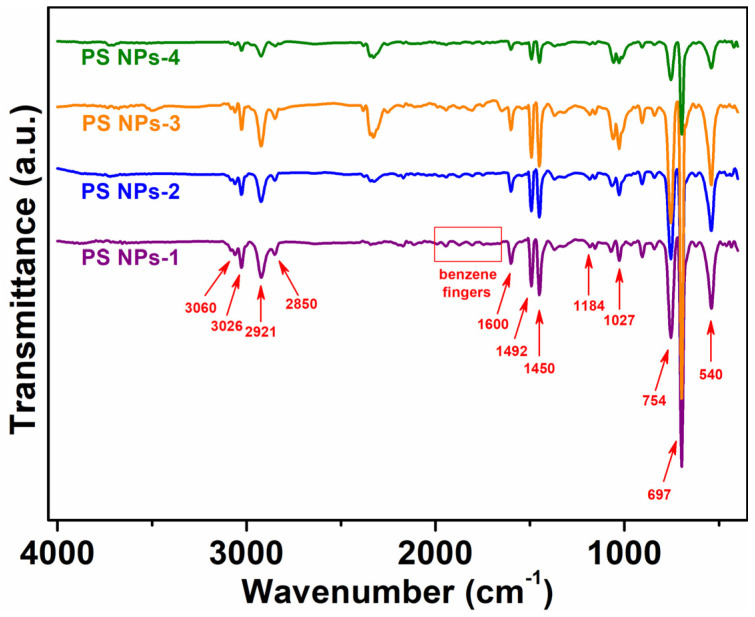
Typical infrared spectra of prepared PS NPs.

The chemical structure of the nanoparticles was investigated by means of infrared spectroscopy in ATR mode. The stacked infrared spectra and the band values for all investigated samples are shown in Figure 2. The unsaturated and saturated C–H stretches of polystyrene in the frequency range between 3150 and 2800 cm^−1^ can be easily observed; therefore, the stretching modes of aromatic C–H bonds are detected above 3000 cm^−1^, at 3060 cm^−1^ and 3026 cm^−1^, whereas the asymmetric and symmetric CH_2_ stretches fall below 3000 cm^−1^, at 2921 cm^−1^, and 2850 cm^−1^, respectively [31,32]. Following the decrease in frequency, the benzene fingers of polystyrene are revealed as four infrared bands with reduced intensity between 2000 and 1650 cm^−1^. According to the literature, a pattern of bands established by four evenly spaced benzene fingers is associated with a mono-substituted benzene ring [31]. The infrared bands at 1600 cm^−1^ and 1492 cm^−1^ may be correlated with the often-called “ring modes” of benzene. The latter originates from the stretching modes of C=C bonds in an aromatic ring [31,33]. At the same time, the infrared band around 1450 cm^−1^ is specific to aliphatic C–C-type bonds [32]. The biggest infrared bands in the spectrum are detected at low frequencies, between 800 and 500 cm^−1^, being attributed to aromatic CH out-of-plane deformation bending [31,32]. Additionally, the S–O stretching bands around 1184 cm^−1^ (broad, asymmetric mode) and 1027 cm^−1^ (sharp, symmetric mode) [34] indicate the presence of the NaVBS component in the chemical structure of nanoparticles. Note that the intensities of these bands are relatively low as compared to those of polystyrene components and that is because of the low content of -SO_3_^−^ functional groups from the NaVBS “surfmer”.

### 3.3. Morphology

**Figure 3:**
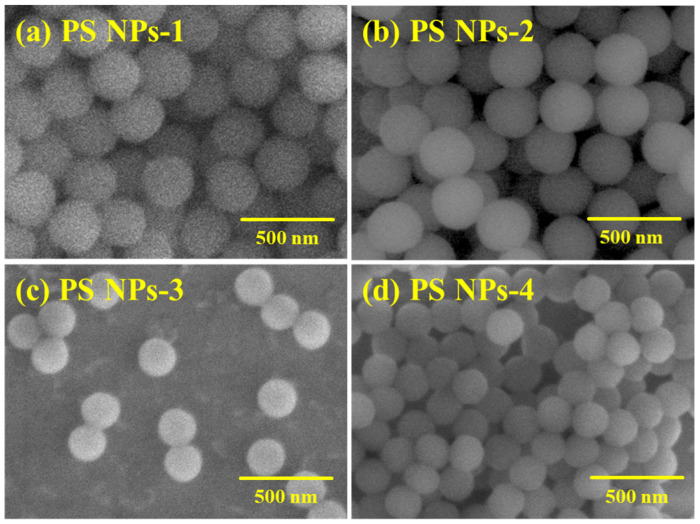
SEM images of (**a**) PS NPs-1, (**b**) PS NPs-2, (**c**) PS NPs-3, and (**d**) PS NPs-4.

A few representative SEM images of the polystyrene nanoparticles are presented in Figure 3. We observe that the nanoparticles are spherically shaped with uniform sizes, evidence that the synthesis conditions were accurately optimized. At the same time, it is evident that the nanoparticles are well distributed onto the aluminum sample holder, suggesting that the aqueous nanoparticle suspension was appropriately dried and the purification of final products after synthesis of nanoparticles was successfully performed.

### 3.4. Thermal Stability

**Figure 4:**
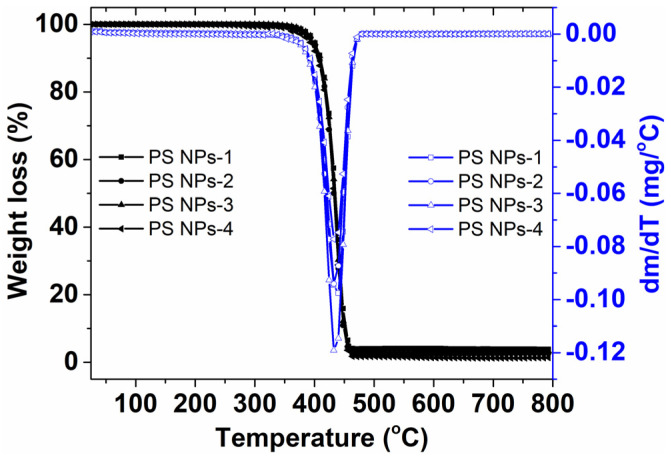
Graph showing the thermogravimetric and the first derivative of the thermogravimetric curve for prepared PS NPs.

The amount of change in the mass of PS NPs as a function of temperature is shown in Figure 4 for PS NPs samples. The TG thermogram for PS NPs-1 exhibits a single loss stage, with the start temperature of the degradation process at 408 °C and the temperature of the complete degradation process at 450 °C. The maximum degradation temperature is found to be at about 436 °C. The results are representative of all the investigated samples. This observation obviously certifies the high degree of stability for our PS NPs. Note, the most common situations in the literature reported that PS NPs exhibit a thermal degradation between 227 °C and 425 °C [35,36]; however, it is clear that characteristic thermal degradation of a material depends on the polymerization method, synthesis conditions, physical parameters used for thermal analysis, etc. For instance, Davodi and coworkers prepared PS NPs by microemulsion polymerization of styrene, including APS as the oxidant and PVP as the surfactant. They finally obtained spherical nanoparticles with diameters around 170–190 nm and the T_onset_ about 261 °C [37]. In our case, the high degree of crosslink is reflected in a superior T_onset_ value.

### 3.5. Glass Transition Temperature

**Figure 5:**
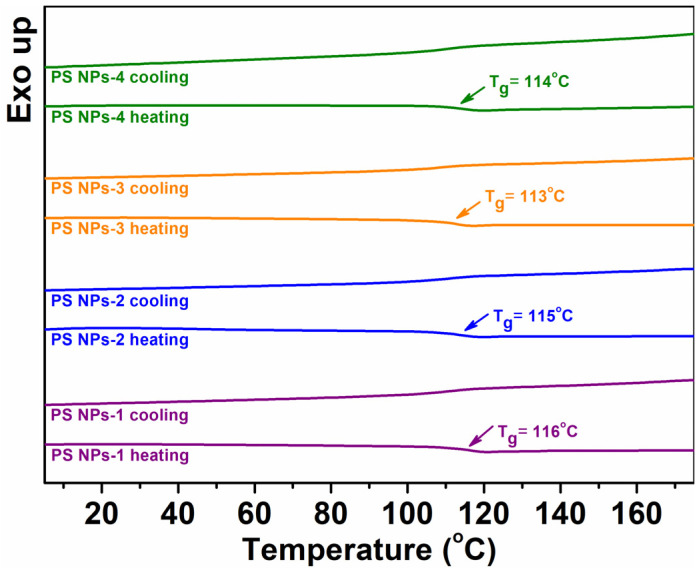
Representative DSC thermograms for PS NPs during second heating–cooling cycles.

Figure 5 shows the second heating–cooling DSC curves for all PS NPs samples. Regarding the second heating regime, the main thermal event is the glass transition (T_g_) step above 113 °C, corresponding to the polystyrene core [38]. We note that the synthesis parameters modify the T_g_ values of final products by ~3 °C, affecting the motions of the mobile chain segments in the PS NPs. These effects will be discussed later, in comparison with the corresponding dielectric spectroscopy data. As observed, no crystallization exothermal signal and neither endothermic correlations to the melting temperature are found, revealing that the PS NPs are entirely amorphous polymers [39].

### 3.6. Assessment of the Dielectric Behavior of Polystyrene Nanoparticles

#### 3.6.1. Evolution of Dielectric Permittivity with Frequency

**Figure 6:**
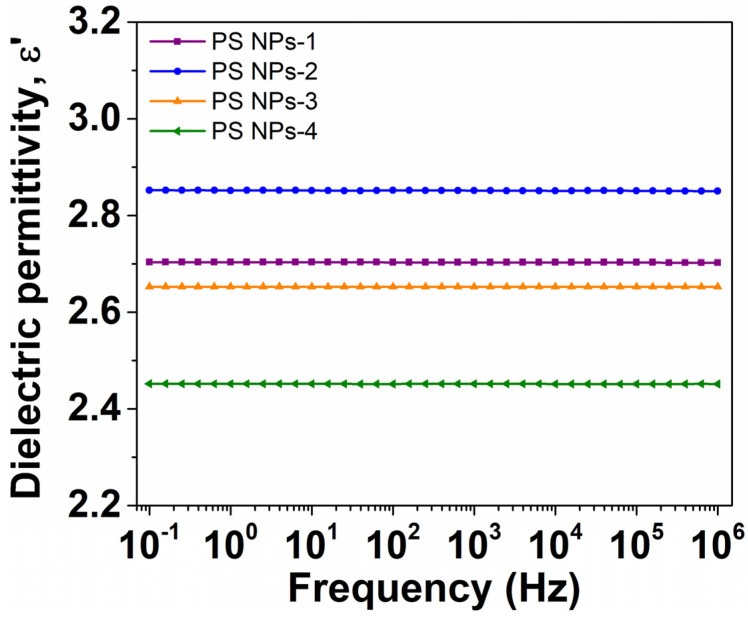
The evolution of dielectric permittivity with frequency at room temperature for PS NPs.

The behavior of measured permittivity, ε′, with frequency at room temperature is displayed in Figure 6 for four polystyrene samples. It is well known that, commonly, the dielectric permittivity decreases gradually towards increasing frequency due to the phase lag between the oscillations of an external alternating electric field and the orientation of dipole moments that are retrieved in the bulk material [40]. The drop of ε′ is more pronounced at low frequencies, while it becomes modest, even negligible, in the high-frequency limit. The latter corresponds to ε_∞_ and may be associated with the ‘intrinsic’ dipolar activity recorded in the bulk [41]. In our case, the dispersion magnitude of PS NPs is moderately reduced with the frequency increase, suggesting a low dipolar activity. This effect is characteristic of non-polar polymers, such as in our case, whose ε′ remains nearly frequency-independent in the entire frequency range. The numerical values of ε_∞_ (Table 2 at 100 kHz) are relatively low, between 2.45 (PS NPs-4) and 2.85 (PS NPs-2), being comparable with polystyrene reported in the literature [42,43]. We will point out later the connection between ε_∞_ and the parameters of synthesis.

**Figure 7:**
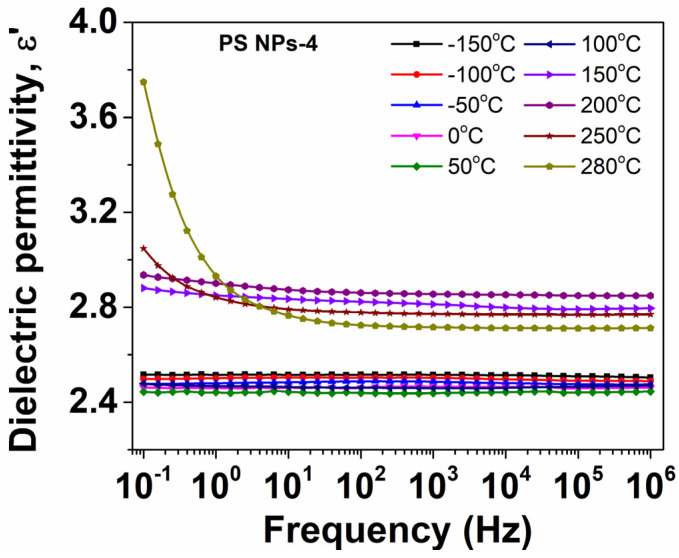
Evolution of dielectric permittivity with frequency at various temperatures for PS NPs.

In Figure 7, the isothermal plots of dielectric permittivity as a function of frequency at various temperatures are exemplarily presented for the PS NPs-4 sample. At low temperatures, ε′ presents a slight decrease with increasing frequency, revealing the ‘intrinsic’ dipolar activity (ε_∞_) of the material. At high temperatures, however, a significant drop of ε′ is provided at low frequencies. This dispersion is due to the thermal activation of charge carriers [25].

#### 3.6.2. Evolution of Dielectric Permittivity with Temperature

**Figure 8:**
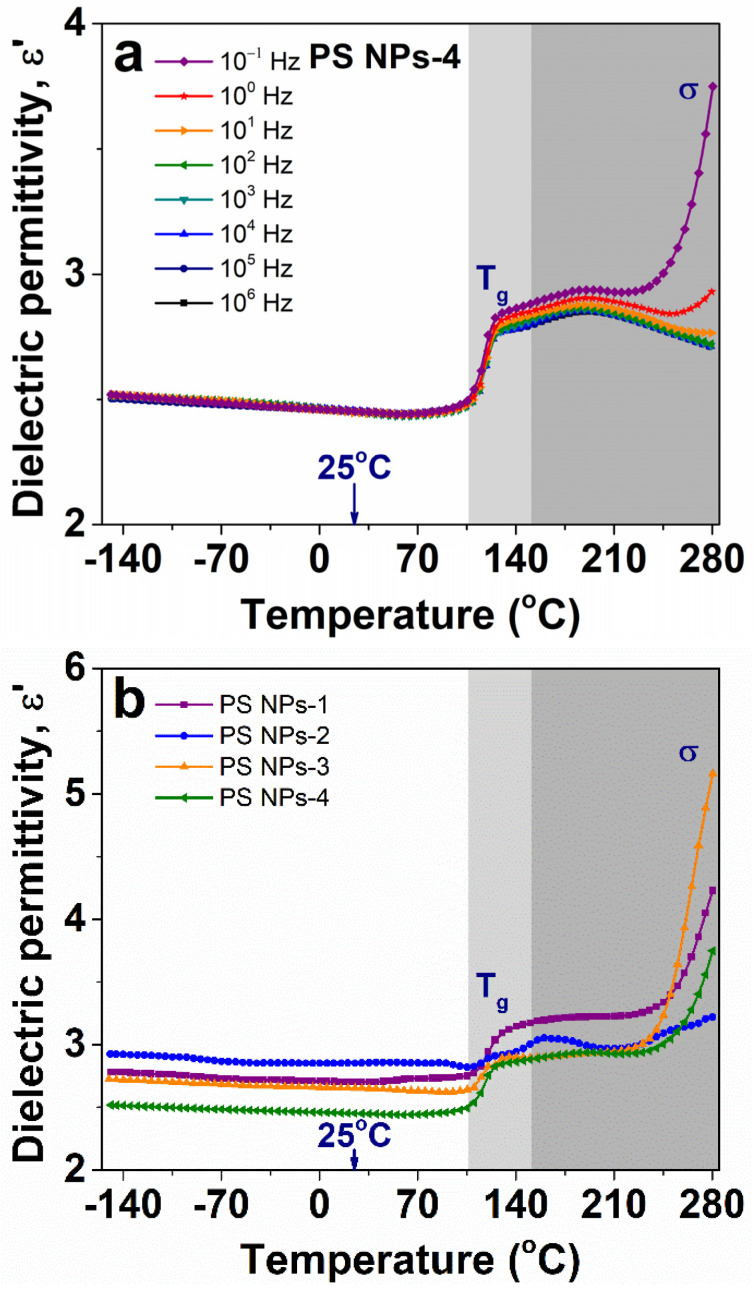
(**a**) Evolution of dielectric permittivity with different temperatures at various frequencies for PS NPs-4 sample, and (**b**) comparative evolution of dielectric permittivity with temperature at 0.1 Hz for all investigated samples. The light grey marked area delimitates the step-increase in ε′ when the glass transition of polystyrene occurs. The darker grey marked area corresponds to the increase in ε′ as a consequence of the existence of free charges.

Figure 8a shows representative temperature dependencies of measured permittivity at various frequency curves for PS NPs-4. At temperatures between −150 °C and 110 °C, the nanoparticles absorb a reduced quantity of thermal energy and, consequently, a limited number of polarizable units are sensitive to the oscillations of the electrical field. Accordingly, we may highlight that the material presents excellent thermal stability, the latter being necessary for engineering materials that operate in a large temperature range. At the same time, we notice a slight decline tendency in ε′ towards increasing temperature. Recalling the data from the literature, H. Sasabe et al. studied the effects of temperature and pressure on the ε′ parameter in non-polar polymers such as polyethylene, poly(tetrafluoroethylene), polypropylene, and polystyrene. Starting with the premise that non-polar dielectric polymers have no permanent dipoles and that only the electronic and atomic polarizations are activated by an applied external electric field, it was found that the value of permittivity is seriously influenced by the material density; moreover, they found that the variation in ε′ with temperature and pressure is much smaller than that of density in the available temperature and pressure ranges. The effect is caused by distortions in the electron clouds that surround the atomic nuclei [41]. Their founding correlates well with the dielectric spectra of PS NPs in Figure 8a.

Following the temperature increment, the step-increase in ε′ rising around 110 °C is connected with the glass transition temperature (T_g_) of PS NPs. We can see here that, in contrast to the declining trend observed at low temperatures, above T_g_, ε′ is gradually enhanced towards increasing temperatures. The latter variation is mainly governed by large groups or segments from macromolecular chains that have received sufficient thermal energy and, as a result, have become sensitive to temperature changes [25]. With a further increase in temperature, the dielectric response is dominated by a sharp increase in ε′, especially for temperature dependence recorded at high frequencies. This behavior is generally assigned with the transport of free charge carriers through the polymer backbone [25].

As shown in Figure 8b, all the investigated PS NPs exhibit similar behavior of ε′ with temperature. Furthermore, two major regions may be considered, as follows: (i) a low-temperature region with a modest dipolar activity; and (ii) a high-temperature region enclosing the glass transition and the charge transport processes, with a noticeable enhancement of ε′ with the increase in temperature.

Next, we turn our attention to the interconnection between thermal characteristics and dielectric measurements of PS NPs. In the recent past, Serghei and coworkers used dielectric data to investigate the phase transitions of ferroelectric polymer nanowires [29]. More precisely, by performing the first derivative of the dielectric permittivity parameter $\left(\frac{\partial \varepsilon^{\prime }}{\partial T}\right)$, they assessed the density fluctuations and hence the phase transition behavior of polyvinylidene fluoride-co-trifluoroethylene moiety. Accordingly, they detected the crystallization, melting, and ferroelectric Curie transitions in excellent correlation with the DSC measurements.

**Figure 9:**
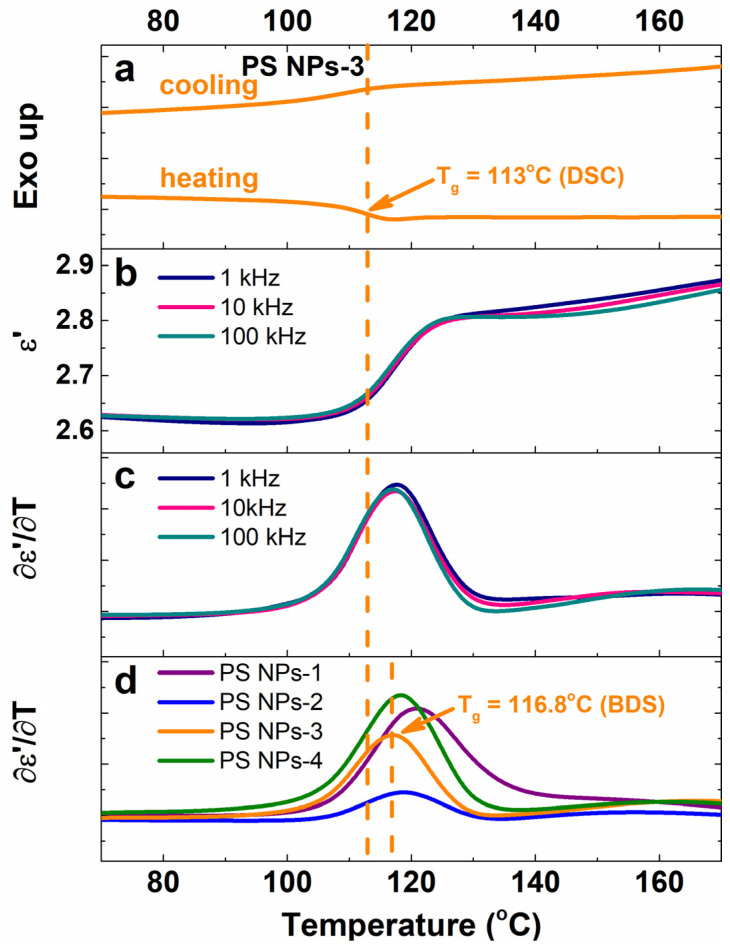
(**a**) Representative DSC thermograms during second heating–cooling cycles; (**b**) isochronal plots of ε′ as function of temperature at various frequencies; (**c**) first derivative of ε′ as function of temperature at various frequencies; and (**d**) first derivative of ε′ as function of temperature at 100 kHz for all investigated samples. The calorimetric and dielectric data retrieved in (**a**–**c**) are selected for NPS-3 nanoparticles. The vertical dash line marks the glass transition of PS NPs-3 sample recorded by DSC and BDS measurements.

Taking into consideration the latter approach, in this section we intend to use the permittivity data to estimate the glass transition temperature of PS NPs. The calorimetric and dielectric data for PS NPs-3 nanoparticles are shown in Figure 9a–c. Then, in Figure 9d, the derivative of permittivity with respect to temperature $\left(\frac{\partial \varepsilon^{\prime }}{\partial T}\right)$ for all investigated nanoparticles measured upon heating is comparatively displayed for all investigated samples. The signal of the glass transition temperature appears as a step-reduction in the DSC heating curve (Figure 9a) and an obvious increase in the magnitude of ε′ (Figure 9b). Following the first derivative of permittivity data, a dominant peak attributed to glass transition is retrieved. We notice that the dielectric T_g_ is detected at a higher temperature than the corresponding calorimetric analog. This finding is somehow expected because, in dielectric spectroscopy, a dielectric peak related to a relaxation process occurs at higher temperatures with the frequency increase. For example, the dielectric T_g_ is found at 115.2 °C at 0.1 Hz and 116.8 °C at 100 kHz; however, as Serghei and coworkers mentioned, to evaluate the dielectric T_g_, the dielectric data are worth selecting in the high-frequency limit, which is not affected by dielectric dispersions (such as electrode polarization and interfacial polarization effects) [29]. The numerical values of T_g_ were associated with the temperatures at which the peak maxima occur.

#### 3.6.3. Synthesis Parameters and Physical Properties

The final analysis refers to the connection between the synthesis factors and the thermal and dielectric properties of the final products. The increase in DVB amount leads to a higher crosslink density. The synthesis parameters as well as the physical properties of the crosslinked spherical PS nanoparticles are listed in Table 1 and Table 2, respectively. It is important to mention that in order to increase the accuracy of experimental results, each material was measured multiple times and the average values were taken into consideration. Since the amount of DVB is slightly varied, no major differences in the physical properties of nanoparticles are expected; however, we may reveal some interesting changes in nanoparticles’ diameter, glass transition, and permittivity.

The series of PS NPs can be divided into two categories, namely PS NPs-1 and PS NPs-2 with a low amount of NaVBS (78 mg) and PS NPs-3 and PS NPs-4 with a large amount of NaVBS (200 mg). The primary function of NaVBS is as an emulsifier, and normally using less of it will result in a larger average of NPs being generated, or fewer nucleation micelles [44]. Additionally, a smaller average dimension of PS NPs will result from the higher fraction of DVB. As the volume of DVB is raised from 0.81 mL to 1 mL, the average diameter of nanoparticles decreases from 286 nm to 178 nm, as shown in Table 1 and Table 2. This finding shows that synthesis factors may accurately control the diameter of nanoparticles. This latter point is very important in the subsequent use of these products as seeds to prepare amphiphilic and snowman-shaped nanoparticles, in which their diameter plays an important role.

Another observation is that higher crosslinking density shifts the glass transition to higher values of temperature as expected but this observation is true when comparing the PS NPs within the same category, PS NPs-1 vs. PS NPs-2 and PS NPs-3 vs. PS NPs-4, Table 2. In other words, this suggests a harder diffusion of macromolecular chains in the nanoparticles. In this case, the crosslinking effect is expected to intensify and, consequently, increase the T_g_ of PS NPs-1 and PS NPs-2 as compared to other examined samples prepared with 200 mg NaVBS.

Last but not least, it is significant to note that the crosslinking agent gradually lowers the dielectric permittivity of the prepared nanoparticles from 2.85 to 2.45 (see Table 1 and Table 2). We believe that the decreased value of ε_∞_ may be due to the increased rigidity of the amorphous backbone, which reduces the molecular mobility of polarizable units.

## 4. Conclusions

Polystyrene nanoparticles were successfully synthesized by emulsion polymerization of styrene in the presence of different amounts of co-polymerizable NaVBS surfactant monomer and DVB crosslinking agent. The nanoparticles are spherical, highly monodisperse, and reveal a high degree of thermal stability, with the maximum degradation temperature found around 436 °C. The latter is noteworthy since it outperforms the thermal stability of other polystyrene-type nanoparticles reported in the literature. The morphology of the nanoparticles is not noticeably affected by the amount of NaVBS and DVB agents, even when the “surfmer” is used in the large amount of 200 mg; however, we found that the increase in the DVB and NaVBS leads to a considerable reduction in the average diameter as well as the ‘intrinsic’ dielectric permittivity of nanoparticles. An interesting result was revealed by BDS after the analysis of a series of PS NPs with varying amounts of DVB and NaVBS, which is the possibility to predict their glass transition temperature by performing the first derivative of isochronal plots of dielectric permittivity as a function of temperature. The DSC measurements validated this procedure. We further observed that the increase in DVB content shifts the glass transition to higher temperature values, restricting the mobility of macromolecular chains in nanoparticles. Future work will focus on the preparation of the next generation of two-lobe Janus-type fillers from these seed PS NPs, with adjustable dielectric properties and enhanced dispersibility in an aqueous solution as a green platform for the preparation of nanocomposites.

## Figures and Tables

**Table 1:** Series of PS NPs synthesized with varying diameters and compositions, mass, and volume of monomers used (m_NaVBS_, V_Styrene_, and V_DVB_).

Sample	m_NaVBS_ (mg)	V_Styrene_ (mL)	V_DVB_ (mL)
PS NPs-1	78	25	0.81
PS NPs-2	78	27	0.81
PS NPs-3	200	26.2	0.85
PS NPs-4	200	26.2	1.0

**Table 2:** Physical (diameter, T_g_ (DSC), T_g_ (BDS), and ε_∞_) parameters for the series of PS NPs synthesized. The T_g_ values obtained by BDS were retrieved at 100 kHz. Values of ε_∞_ are selected at 1 Hz and 25 °C.

Sample	Mole Fraction of Crosslinker (%)	Diameter (mm)	T_g_ (DSC) (°C)	T_g_ (BDS) (°C)	ε_∞_
PS NPs-1	2.536	271	116	120.8	2.7
PS NPs-2	2.353	286	115	118.7	2.85
PS NPs-3	2.533	191	113	116.8	2.65
PS NPs-4	2.967	178	114	118.2	2.45

## Data Availability

Not applicable.
